# Analysis of the Rate of Re-planning in Spot-Scanning Proton Therapy

**DOI:** 10.14338/IJPT-21-00043.1

**Published:** 2022-06-28

**Authors:** Yue-Houng Hu, Riley H. Harper, Noelle C. Deiter, Jaden D. Evans, Anita Mahajan, Jon J. Kruse, Daniel W. Mundy

**Affiliations:** 1 Department of Radiation Oncology, Division of Medical Physics, Mayo Clinic, Rochester, MN, USA; 2 College of Science and Engineering, University of Minnesota, Minneapolis, MN, USA; 3 Department of Radiation Oncology, Mayo Clinic, Rochester, MN, USA; 4 Department of Radiation Oncology and Precision Genomics, Intermountain Cancer Center, Ogden, UT, USA

**Keywords:** IMPT, re-planning, verification scanning

## Abstract

**Purpose:**

Finite proton range affords improved dose conformality of radiation therapy when patient regions-of-interest geometries are well characterized. Substantial changes in patient anatomy necessitate re-planning (RP) to maintain effective, safe treatment. Regularly planned verification scanning (VS) is performed to ensure consistent treatment quality. Substantial resources, however, are required to conduct an effective proton plan verification program, which includes but is not limited to, additional computed tomography (CT) scanner time and dedicated personnel: radiation therapists, medical physicists, physicians, and medical dosimetrists.

**Materials and Methods:**

Verification scans (VSs) and re-plans (RPs) of 711 patients treated with proton therapy between June 2015 and June 2018 were studied. All treatment RP was performed with the intent to maintain original plan integrity and coverage. The treatments were classified by anatomic site: brain, craniospinal, bone, spine, head and neck (H&N), lung or chest, breast, prostate, rectum, anus, pelvis, esophagus, liver, abdomen, and extremity. Within each group, the dates of initial simulation scan, number of VSs, number of fractions completed at the time of VS, and the frequency of RP were collected. Data were analyzed in terms of rate of RP and individual likelihood of RP.

**Results:**

A total of 2196 VSs and 201 RPs were performed across all treatment sites. H&N and lung or chest disease sites represented the largest proportion of plan modifications in terms of rate of re-plan (RoR: 54% and 58%, respectively) and individual likelihood of RP on a per patient basis (likelihood of RP [RP%]: 46% and 39%, respectively). These sites required RP beyond 4 weeks of treatment, suggesting continued benefit for frequent, periodic VS. Disease sites in the lower pelvis demonstrated a low yield for RP per VS (0.01-0.02), suggesting that decreasing VS frequency, particularly late in treatment, may be reasonable.

**Conclusions:**

A large degree of variation in RoR and individual RP% was observed between anatomic treatment sites. The present retrospective analysis provides data to help develop anatomic site–based VS protocols.

## Introduction

While finite proton range potentially allows for superior dose conformality in comparison to other modalities of radiation therapy, delivery is inherently sensitive to changes in patient geometry. Several approaches to adaptive radiation therapy have been proposed to address changes in patient anatomy throughout the course of proton treatment. Adaptive therapy solutions are often categorized as either online [1]—where plans may be adjusted at the time of treatment, based on daily imaging (typically treatment image guidance) to account for interfraction changes in patient anatomy—or offline, where plans may be adapted, based on periodic imaging acquired between treatments [2]. Further, changes in patient anatomy may, to some extent, be addressed in treatment planning and quality assurance, optimizing and evaluating plans for robustness to some degree of geometric uncertainty [3–5].

Using an offline adaptive therapy model, precise setup and careful monitoring of the patient habitus is a necessity for safe treatment delivery. One such process includes periodic verification scanning (VS) using computed tomography (CT) imaging. While this method affords accurate, periodic reconstruction of the patient anatomy, each VS occupies a substantial quantity of clinical resources, requiring allocation of time for the CT scanner and clinical personnel, potentially including radiation therapists, medical physicists, medical staff (eg, medical doctors, nurse practitioners, and/or physician assistants), and medical dosimetrists. Optimization of VS interval would afford sufficient information about the rate of change of patient anatomy for potential clinical intervention (eg, re-planning [RP]) while also minimizing usage of institutional resources for the scanning program.

To date 3 clinical, retrospective studies have been published on this topic. One, a 6-patient study for head and neck (H&N) intensity-modulated proton therapy (IMPT), determined that the optimal timing for offline adaptive RP is 4 weeks [6]. The second, a follow-up, 10-patient, oropharyngeal study found that after the fourth week of verification imaging, mean volumes of clinical target volumes and parotid glands significantly decreased. Further, mean doses to clinical target volumes decreased and mean doses to parotid glands and oral cavities increased [7]. Finally, a 160-patient study was conducted to determine the statistically correlated factors that may be predictive of H&N IMPT RP [8].

To help address the deficiency in available VS and RP data, the present study analyzes patient data from a variety of treatment sites, including brain, craniospinal, bone, spine, H&N, lung or chest, breast, prostate, gynecologic region, rectum, anus, pelvis, esophagus, liver, abdomen, and extremity. Within each anatomic site studied, a variety of data were collected, including date of initial simulation, number of VSs, number of fractions completed at time of VS, and frequency of RP. The data were analyzed in terms of rate of RP (RoR) and individual patient likelihood of RP (RP%).

## Materials and Methods

All data were collected in accordance with procedures outlined in Study 18-004295, approved by the Mayo Clinic Institutional Review Board. A waiver of written informed consent was granted on the basis of the retrospective nature of the study and absence of impact on patient care.

### Treatment Planning

All treatment plans were robustly optimized by using the Eclipse treatment planning system (Varian Medical Systems, Palo Alto, California). Unless otherwise noted, robustness was evaluated with a 3% range uncertainty and 3-mm setup uncertainty. Typically, a general planning criterion of 95% target coverage by the 95% prescription isodose line is used but is subject to other planning considerations. Motion mitigation strategies for sites susceptible to breathing motion include layer-by-layer repainting, passive breath holding, and phase gating. In general, repainting is used when target motion exceeds 5 mm and breath hold or phase gates (defined by external surrogate motion) are set larger than 1 cm. These are not strict constraints and motion mitigation may be implemented for cases with smaller target motion, based on dosimetric analysis. For plans using posterior beams, patients are typically positioned atop a low-density foam pad to improve patient comfort and smooth the posterior external contour, subsequently improving the reproducibility of patient position.

#### Head and neck

Patients are immobilized on an Orfit HP Pro couch using an Orfit thermoplastic mask (Orfit Industries NV, Wijnegem, Belgium) and custom Klarity head cushion (Klarity Medical Products, Heath, Ohio). Multifield optimization (MFO) is used to better reduce exposure to organs at risk (OARs) and maximize target coverage. Standard beam arrangements for bilateral H&N use 2 anterior oblique beams and 1 posterior beam. Alternative geometries may be used as necessary, for example, to avoid treating through dental hardware.

#### Spine

For spine treatment sites, patients are typically immobilized in the same fashion as for H&N treatment sites. The standard treatment technique recommends single-field optimization (SFO) planning and 2 posterior oblique fields with the possible addition of a straight posterior field. MFO planning is an acceptable alternative, especially in cases with spinal hardware.

#### Craniospinal Irradiation or Brain

Craniospinal irradiation (CSI) patients are immobilized in the same fashion as for H&N treatment sites. Beam arrangements have evolved with time and experience with the current standard-of-practice using a series of posterior fields overlapping along the length of the brain and spine with an adjunct superior oblique field covering to the inferior edge of the brainstem. Sufficient field overlap and interfield robust optimization of the spine fields generates a smooth dose gradient between fields of ∼1%/mm.

For brain treatment sites, patients are immobilized similarly, additionally including Orfit HP Pro couch, thermoplastic mask, and Klarity head cushion. Typically, 3 to 4 fields with hinge angles >30° are used, avoiding fields with end ranges abutting the brainstem, optic nerves, and chiasm. Beams entering through the eyes or parallel to the ear canals are also avoided.

#### Breast

For smaller targets, including partial breast irradiation (PBI), SFO is used to improve plan robustness. For chest wall and whole breast plans, MFO is typically used to improve OAR sparing and to accommodate fields not irradiating the entirety of the volume. Patients are typically set up on a supine breast board with a head rest and open face mask. Planning may be optionally completed with arms up or down. Additionally, non-PBI treatments are evaluated at 3% range uncertainty and 5-mm setup uncertainty for robustness.

#### Esophagus

For esophagus treatment sites, patients are immobilized with arms up in an upper vacuum bag and indexed knee cushion. This site is commonly treated with SFO but may also be treated with MFO upon physician request or for normal tissue sparing. The standard beam arrangement is 2 posterior oblique beams. Robustness is assessed for 5-mm shifts and 3% proton range uncertainty.

#### Gynecologic, rectal, anal

For gynecologic, rectal, and anal treatment sites, patients are immobilized with a lower vacuum bag or knee cushion. SFO is the default technique but switching to MFO techniques is common to better achieve planning objectives. Beam selection may vary from 2 to 3 fields consisting of 2 posterior oblique beams and 1 posterior beam, or 2 posterior oblique beams and 1 anterior beam for cases with inguinal node involvement. Robustness is assessed for 5-mm shifts and 5% range uncertainty.

#### Liver

For liver treatment sites, patients are immobilized with arms up in an upper vacuum bag and indexed knee cushion or lower vacuum bag. SFO or MFO technique may be used depending on skin or normal tissue sparing. Beam selection commonly includes 2 to 3 fields biased towards patient's right side. Robustness is evaluated for 5-mm shifts in the lateral and anterior-posterior directions, 7 mm in the superior-inferior direction, and 3% proton range uncertainty.

#### Lung or chest

For lung or chest treatment sites, patients are immobilized with either arms up in a vacuum bag or with arms down and a 5-point thermoplastic mask, depending on target location and patient tolerance. These sites are commonly treated with SFO technique unless split scanning target volumes [9, 10] are required or improved OAR sparing is necessary. Beam selection may vary depending on location of target, but typically includes 2 to 3 fields. Robustness is assessed for 5-mm shifts and 3% proton range uncertainty.

#### Prostate

For prostate treatment sites, patients are immobilized with an indexed knee cushion and/or lower vacuum bag. This site is commonly treated with the SFO technique. Beam selection includes 2 lateral beams or 2 lateral and 2 anterior oblique beams.

### Analysis

Throughout the course of a given proton treatment, VSs are periodically scheduled (approximately weekly) to monitor potential gross changes in patient anatomy. VS data are registered to original planning scans and dose from the original plan is calculated on the current anatomy. Typically, normal structures, OARs, and any other structures with overridden HU values relevant to planning are rigidly registered to the VS. Target volumes are transferred to VS via rigid and deformable registration with the intention of guiding the review process. Deformable registrations are not presented to provide accurate quantification of dose statistics. Although dose-volume histogram metrics are suggested to guide the decision-making process with respect to RP (eg, maintain 95% dose to 95% of the target volume), the treating physician ultimately retains discretion, and decisions are based primarily on a slice-by-slice review of the VS dose distribution. Specific reasons for RP vary by disease site but are typically associated with decreased target coverage or increased OAR dose due to variations in patient anatomy relative to the planning CT. Variation in target size throughout the course of treatment might also trigger an RP, but most are completed with the intent to maintain the integrity of the original treatment plan.

At the author's institution, data were collected for patients who received treatments between June 2015 and June 2018. All data were collected via extraction from the electronic medical record system and categorized, primarily by treatment site, including brain (63); craniospinal region (26); bone or spine (38); H&N (169); lung or chest (38); breast (121); prostate (138); gynecologic region, rectum, anus (18); pelvis (14); esophagus (35); liver (18); abdomen (33); and extremity (1). The data from 711 patients were analyzed, representing a total of 1272 VSs and 198 RPs.

Metrics including RoR and RP% were calculated to determine the clinical burden of RP. RoR was defined as:



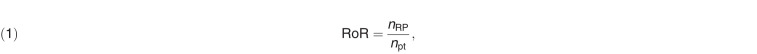

where *n*_pt_ and *n*_RP_ are the total count of patients and RPs, respectively, and represents the global value of the number of RPs per patient. RP% was quantified as:



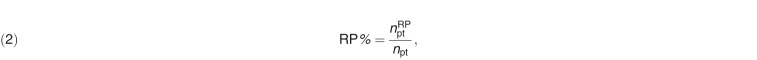

where 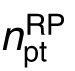 was defined as the number of patients whose course of treatment included at least 1 RP. Thus, RP% represents the likelihood of RP for any given patient's treatment course. RoR may be greater than or equal to RP%. RoR may be higher than RP%, since a patient's treatment course may include multiple RPs.

## Results

**Figure 1.**
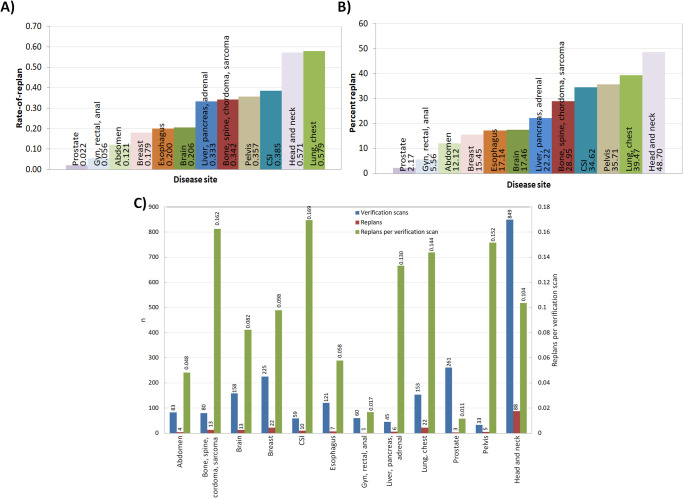
Bar plots of RoR (A) and RP% (B) and a dual axis bar plot (C) of the total number (n: plotted along the primary vertical axis) of VSs (blue) and RPs (red) as well as RPs per VSs (green, plotted along the secondary vertical axis) all categorized by treatment site. Numerical values for each quantity in (A) and (B) are stated directly on the bar graph. Abbreviations: CSI, craniospinal irradiation; RoR, rate of re-plan; RPs, re-plans; RP%, individual likelihood of replan; VSs, verification scans.

**Figure 1** displays bar plots of the observed RoR (**Figure 1A**) and RP% (**Figure 1B**) categorized by treatment site and a comparison of quantities (n) of VS to RPs, as well as the number of RP per VS on a dual-abscissa plot (**Figure 1C**). H&N and lung or chest treatment sites represent the greatest encumbrance in terms of RoR and RP%. Although lung or chest plans exhibit a higher RoR, head and neck patients are individually more likely to require RP. Over all treatment sites, each VS finding has an approximately 10% likelihood of generating an RP, with the largest probability being observed for craniospinal as well as bone, spine, chordoma, and sarcoma sites.

**Figure 2.**
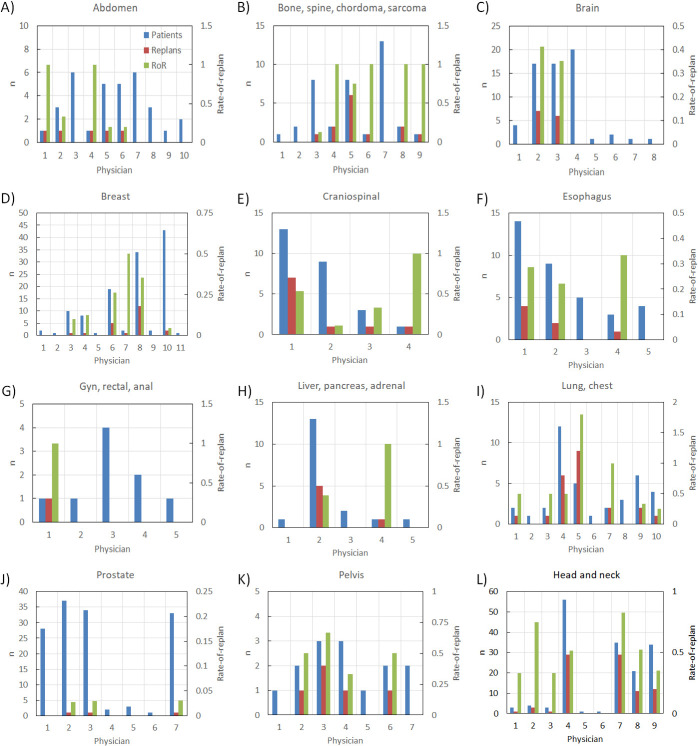
Dual axis bar plot visualizing comparisons of total number (n) of patients (blue) and RPs (red) along the primary (left vertical) axis, and RoR (green) plotted on the secondary (right vertical) axis, separated by treating physician (identified numerically) and categorized by treatment site. Abbreviations: RoR, rate of re-plan; RP, re-plans.

**Figure 2** plots the total number of patients (blue) and RPs (red) along the left vertical axis, and RoR (green) along the right vertical axis separated by physician (identified numerically) for each studied disease site. It should be noted that the physician numbers are assigned in chronological order for each treatment site. Any given physician may participate in treatment of multiple disease sites and may not necessarily be assigned the same identification number over all groups. Mean and standard deviations for RoR are tabulated in the **Table**.

**Table.** Mean RoR% ( 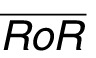) and standard deviation (σ_RoR_) categorized by physician primary disease site.

**Treatment site**	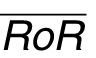	**σ_RoR_**
Abdomen	27.3	40.1
Prostate	1.24	1.55
Bone, spine, chordoma, sarcoma	54.2	49.2
Breast	16.4	18.6
Liver, pancreas, adrenal	27.7	43.7
Lung, chest	48.8	55.6
Pelvis	28.6	28.4
Head and neck	33.1	30.6
Esophagus	16.8	15.8
Gynecologic, rectal, anal	20.0	44.7
Brain	9.56	17.8
CSI	49.6	37.9

**Abbreviations:** RoR, rate of re-plan; CSI, craniospinal irradiation.

**Figure 3.**
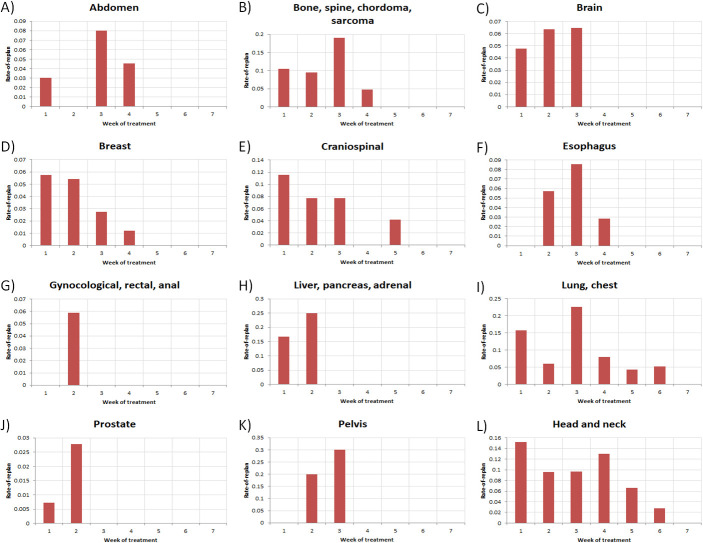
Histograms of RoR as a function of treatment week for each disease site. Abbreviation: RoR, rate of re-plan.

The data were also analyzed for where RP occurred in the course of treatment. **Figure 3** displays a series of histograms, categorized by disease site, of the RoR as a function of treatment week. For all disease sites, RP probabilities were highest within the first 4 weeks of treatment. Re-plans after week 4 were only found in CSI, lung or chest, and H&N disease sites. One RP was observed after week 4 for CSI, as well as 11.4% of cases for lung or chest, and 22.2% for H&N treatments.

**Figure 4.**
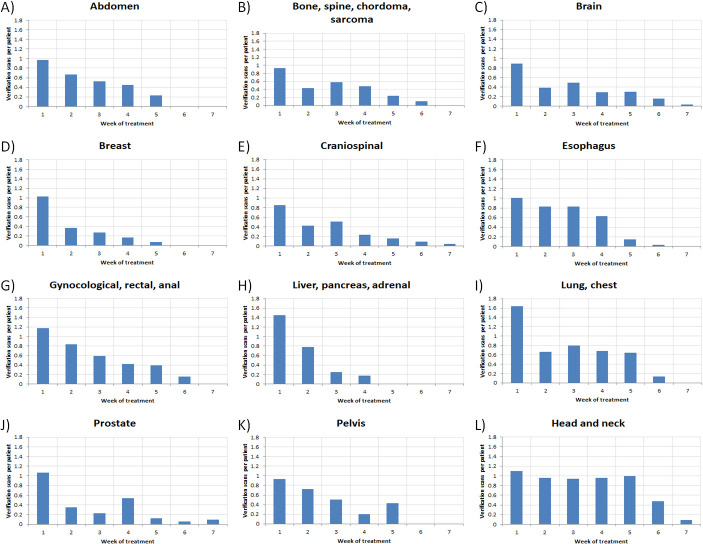
Histograms of the number of VSs per patient as a function of treatment week for each disease site. Abbreviation: VSs, verification scans.

**Figure 5.**
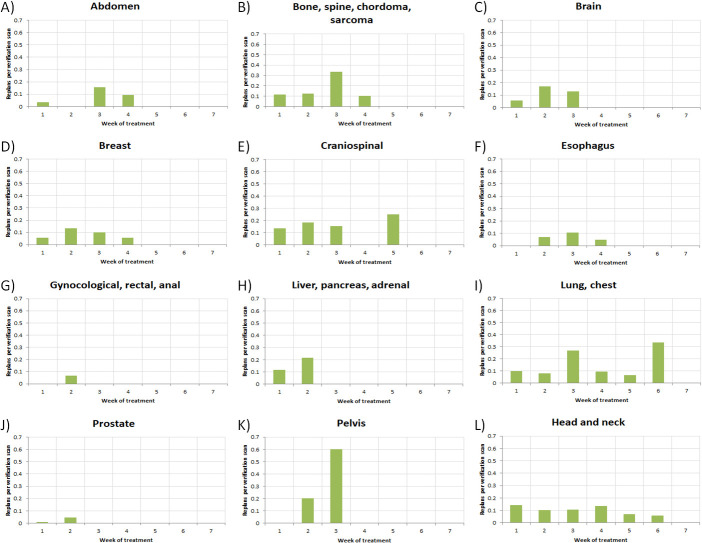
Histograms of RP/VS as a function of treatment week for each disease site. Abbreviation: RP/VS, re-plans per verification scan.

Similarly, **Figures 4** and **5** plot a series of histograms, again categorized by disease site, for VS per patient and for RP per VS, respectively, as a function of treatment week. In general, as seen in **Figure 4**, the number of VSs per patient decreased over the course of treatment. However, in all treatment sites, VSs were acquired at some frequency throughout the duration of treatment. **Figure 5** shows little evidence of a trend in RP per VS as a function of time for any of the disease sites with RP beyond week 4.

## Discussion

Throughout the course of the study period, the H&N disease site represented the largest clinical encumbrance in terms of gross number of RPs with 91. Normalized by number of patients (**Figure 1A** and **1B**), the lung or chest disease site slightly exceeded H&N in terms of RoR, whereas RP% was highest for H&N treatments. Thus, while patients will more typically need RPs with H&N treatments, those who do receive them are less likely to need another when compared to lung or chest patients. While also considering **Figures 3**, **4**, and **5**, the data suggest that continued, periodic VS throughout lung or chest, H&N, and potentially CSI treatments, would be a reasonable practice.

Lower pelvic treatment sites, including prostatic, gynecologic, rectal, and anal, exhibited much lower probabilities of RP as a function of VS (**Figure 1C**) as a whole and as a function of time. The low RoR and scarcity of RP after week 2 (**Figure 3**), in spite of continued observation via VS (**Figure 4**) for these disease sites, suggest that a less conservative approach to VS may be warranted, particularly late in treatment. Although the pelvic treatment site exhibits higher RoR and RP% than the aforementioned lower pelvic disease sites, all of the RPs were observed before week 4, suggesting the possibility of discontinuation of VS late in treatment.

While global conclusions may be drawn by collective data by treatment site, some interphysician variation exists (**Figure 2**, **Table**). This variation exists presumably because of physician discretion over RP, though it is unclear from the collected data whether the discrepancy stems from differences in initial planning approach or tolerance for dosimetric change in the VS. A particularly dramatic example is observed when considering the breast treatment site. The data for the 3 most prolific breast physicians—treating 19, 34, and 43 patients—called for RP 5, 10, and 2 times, respectively, resulting in RoR of 26%, 35%, and 4.7%. Such data may be collected for institutions with similar processes and analyzed on a per-physician basis to effectively review best VS practices.

Finally, RP as a function of time, as reported in **Figure 3**, suggests continual changes in patient anatomy and geometry beyond week 4 for lung or chest and H&N disease sites. These late-treatment RPs suggest pragmatism in continued, regular VS throughout the course of treatment.

## Conclusion

A large degree of variation in RoR and individual RP% was observed between treatment sites. The present retrospective analysis presents data on which to design VS protocols on the basis of this treatment site variability. In general, increases in gross RP quantities, RoR, and RP% were affected by treatment site (with H&N and lung or chest treatments accounting for the largest clinical encumbrance of VS and RP) and treating physician.

## References

[1] Chetty IJ, Fontenot J (2017). Adaptive radiation therapy: off-line, on-line, and in-line. *Int J Radiat Oncol Biol Phys*.

[2] Lim-Reinders S, Keller BM, Al-Ward S, Sahgal A, Kim A (2017). Online adaptive radiation therapy. *Int J Radiat Oncol Biol Phys*.

[3] Tryggestad EJ, Liu W, Pepin MD, Hallemeier CL, Sio TT (2019). Managing treatment-related uncertainties in proton beam radiotherapy for gastrointestinal cancers. *J Gastrointest Oncol*.

[4] Yang Z, Zhang X, Wang X, Zhu XR, Gunn B, Frank SJ, Chang Y, Li Q, Yang K, Wu G, Liao L, Li Y, Chen M, Li H (2020). Multiple-CT optimization: an adaptive optimization method to account for anatomical changes in intensity-modulated proton therapy for head and neck cancers. *Radiother Oncol*.

[5] Deiter N, Chu F, Lenards N, Hunzeker A, Lang K, Mundy D (2020). Evaluation of replanning in intensity-modulated proton therapy for oropharyngeal cancer: factors influencing plan robustness. *Med Dosim*.

[6] Palmer M, Jones T, Waddell M, Varghese S, Zhang Y, Amin M, Yeh BK, Zhu R, Dong L, Frank SJ (2012). The optimal timing for off-line adaptive planning for bilateral head-and-neck IMPT is week 4. *Int J Radiat Oncol Biol Phys*.

[7] Wu RY, Liu AY, Sio TT, Blanchard P, Wages C, Amin MV, Gunn GB, Titt U, Ye R, Suzuki K, Gillin MT, Zhu XR, Mohan R, Frank SJ (2017). Intensity-modulated proton therapy adaptive planning for patients with oropharyngeal cancer. *Int J Particle Ther*.

[8] Evans JD, Harper RH, Petersen M, Harmsen WS, Anand A, Hunzeker A, Deiter NC, Schultz H, Jethwa KR, Lester SC, Routman DM, Ma DJ, Garces YI, Neben-Wittich MA, Laack NN, Beltran CJ, Patel SH, McGee LA, Rwigema J-CM, Mundy DW, Foote RL (2020). The importance of verification CT-QA scans in patients treated with IMPT for head and neck cancers. *Int J Particle Ther*.

[9] Meyer J, Bluett J, Amos R, Levy L, Choi S, Nguyen Q-N, Zhu XR, Gillin M, Lee A (2010). Spot scanning proton beam therapy for prostate cancer: treatment planning technique and analysis of consequences of rotational and translational alignment errors. *Int J Radiat Oncol Biol Phys*.

[10] Pugh TJ, Amos RA, John Baptiste S, Choi S, Nhu Nguyen Q, Ronald Zhu X, Palmer MB, Lee AK (2013). Multifield optimization intensity-modulated proton therapy (MFO-IMPT) for prostate cancer: robustness analysis through simulation of rotational and translational alignment errors. *Med Dosim*.

